# Neutrophils in inflammatory bowel disease: disease-promoting versus protective functions

**DOI:** 10.3389/fimmu.2025.1750743

**Published:** 2026-01-15

**Authors:** Shaochen Yu, Mengjie Zhang, Wenlu Niu, Yuting Huang, Ziyue Dou, Beibei Tian, Langlang Yang, Jian Lu

**Affiliations:** 1 Department of Emergency and Critical Care Medicine, Chuzhou Integrated Traditional Chinese and Western Medicine Hospital, Chuzhou, Anhui, China; 2 Department of Gastroenterology, The First Affiliated Hospital of Anhui Medical University, Hefei, Anhui, China

**Keywords:** immune regulation, inflammatory bowel disease, microbiota, mucosal repair, neutrophil, neutrophil extracellular traps, therapeutic target

## Abstract

Inflammatory bowel disease (IBD) is a complex chronic intestinal inflammatory disorder whose pathogenesis involves aberrant interactions between genetic, environmental, microbial, and immune factors. Neutrophils, as key effector cells of innate immunity, are among the first immune cells to infiltrate the inflamed mucosa in IBD, and their role in the disease course is multifaceted. This review systematically elaborates on the dual functions of neutrophils in IBD. On one hand, activated neutrophils act as crucial “destroyers” promoting the initiation and progression of IBD by releasing effector molecules such as reactive oxygen species (ROS), proteases, and neutrophil extracellular traps (NETs), which disrupt the intestinal epithelial barrier, amplify the inflammatory cascade, promote thrombosis, and mediate resistance to corticosteroids and biologics. On the other hand, neutrophils also play key protective roles by efficiently clearing pathogens and apoptotic cells, secreting pro-angiogenic and tissue repair factors, modulating the stem cell microenvironment, and maintaining microbial homeostasis, thereby actively promoting mucosal healing and inflammation resolution. This article also delves into neutrophil heterogeneity, functional plasticity, and their complex interactions with the microbiota, and proposes new precision therapeutic strategies targeting neutrophils. A comprehensive understanding of the dynamic balance and regulatory mechanisms of this “dual-role guardian” will provide new perspectives for researching IBD pathogenesis and innovating treatments.

## 1 Introduction

Inflammatory bowel disease (IBD), primarily comprising Crohn’s disease (CD) and ulcerative colitis (UC), is a refractory intestinal disorder with a globally rising incidence. Its characteristic pathological features include impaired intestinal mucosal barrier function, aberrant immune activation, and chronic relapsing inflammation (1). The exact etiology remains incompletely understood, but it is widely believed that a dysregulated immune response to the gut microbiota in genetically susceptible individuals is a central element (2).

In the intestine, an environment rich with microbial challenges, the innate immune system constitutes the host’s first line of defense. Neutrophils, the most abundant leukocytes in circulation, are characterized by their potent phagocytic and bactericidal capabilities and rapid recruitment to sites of inflammation (3). Traditionally, neutrophils have been viewed as “first responders” in acute inflammation and “executors” of tissue damage. In IBD, massive neutrophil infiltration into the intestinal mucosa forms characteristic pathological changes like crypt abscesses, and their numbers correlate closely with disease activity; hence, they have long been considered drivers of disease progression (4).

With advances in research technologies, the understanding of neutrophil function is undergoing a paradigm shift. Growing evidence indicates that neutrophils are not a homogeneous, short-lived cell population but exhibit significant heterogeneity, functional plasticity, and finely regulated capabilities (5). They are not only front-line “warriors” but also important “messengers” and “engineers,” deeply involved in immune regulation, tissue repair, and homeostasis maintenance. Within the complex inflammatory microenvironment of IBD, neutrophil function presents a profound paradox: they exacerbate mucosal damage while also promoting repair and regeneration (6). The balance of these dual roles directly influences the outcome of IBD and treatment efficacy.

Therefore, this review aims to transcend the traditional perspective and systematically synthesize the dual regulatory roles of neutrophils in IBD. Starting from their basic biological characteristics, it details their recruitment and infiltration mechanisms in the IBD mucosa; it focuses on analyzing their pathogenic roles in barrier disruption, inflammation amplification, and treatment resistance, while also balancing the discussion with their protective functions in clearing infections, promoting repair, and maintaining homeostasis. Finally, integrating new insights into neutrophil heterogeneity and microbial interactions, it prospects future therapeutic avenues targeting neutrophils, hoping to provide a reference for a comprehensive understanding of IBD immunopathology and the development of novel treatment strategies.

## 2 Neutrophils: the dual-role guardian of intestinal immunity

Neutrophils are the body’s first line of defense against invading pathogens. They undergo meticulous development in the bone marrow, differentiating from myeloblasts into mature cells with polymorphonuclear nuclei. Their development is strictly regulated by various transcription factors and cytokines, among which granulocyte colony-stimulating factor (G-CSF) is a key factor promoting neutrophil production and release (7). Neutrophils are not a homogeneous population; recent studies have revealed significant functional plasticity and heterogeneity, allowing them to adjust their phenotype and function based on microenvironmental signals (5).

Under steady-state conditions, neutrophils have a short half-life of about 6–8 hours in the circulation before being cleared via apoptosis by the mononuclear phagocyte system (8). This process is significantly altered in inflammatory states. Upon tissue injury or infection, endothelial cells are activated and express adhesion molecules, initiating the neutrophil recruitment process (9). This multi-step process includes initial contact, rolling, firm adhesion, and transendothelial migration, each step precisely regulated by specific molecular pairs (10).

### 2.1 A diversified antimicrobial arsenal

The antimicrobial mechanisms of neutrophils reflect an evolutionarily sophisticated design. Phagocytosis is the most direct clearance mechanism, initiated by pattern recognition receptors identifying pathogen-associated molecular patterns (11). After engulfment, the phagosome fuses with intracellular granules to form the phagolysosome, where pathogens are efficiently degraded in an acidic environment by various hydrolases (12).

Reactive oxygen species (ROS) production is central to the potent bactericidal activity of neutrophils (13), primarily catalyzed by the NADPH oxidase complex (also known as phox) (14). In the resting state, the components of this complex are dispersed in the cytoplasm and cell membrane; upon activation signals, they rapidly assemble on the membrane, transferring electrons to oxygen to generate superoxide anion (O_2_^-^), which is then converted to hydrogen peroxide (H_2_O_2_) and other highly reactive oxidants (15). In the presence of myeloperoxidase (MPO), H_2_O_2_ reacts with halide ions (e.g., Cl^-^) to generate hypohalous acids; a key example is hypochlorous acid (HOCl), one of the most potent microbial killing agents, which can destroy key macromolecules like proteins and nucleic acids in pathogens through halogenation and oxidation (16). This series of reactions constitutes the famous “respiratory burst” and is the core mechanism of oxidative killing in neutrophils.

The regulation of degranulation is equally precise, reflecting the body’s hierarchical control of the inflammatory response. Intracellular granules in neutrophils are classified into three levels based on their content and release thresholds: readily released secretory vesicles, specific granules requiring stronger stimuli, and azurophilic granules with the highest release threshold. This graded degranulation mode ensures that neutrophils release effector molecules appropriately according to the threat level, clearing pathogens while limiting collateral damage to host tissues (17–19).

### 2.2 NETosis: a double-edged sword

The discovery of Neutrophil Extracellular Trap-osis (NETosis) revolutionized the understanding of neutrophil cell death. At least two NETosis pathways are currently known: classical suicidal NETosis and vital NETosis (20). The former is an NADPH oxidase-dependent programmed cell death process, while the latter allows neutrophils to release NETs while retaining partial cellular functions and viability. Both pathways likely operate under different pathophysiological conditions.

NETs have a complex composition, with a core of citrullinated histones and decondensed chromatin DNA forming a fibrous network (21). This network is studded with various antimicrobial proteins from different granules, constituting an efficient “targeted delivery” system. For instance, MPO and neutrophil elastase (NE) from azurophilic granules are key components; NE can degrade bacterial virulence factors, while MPO maintains high local levels of hypochlorous acid production within NETs (22). Lactoferrin from specific granules chelates iron ions, inhibiting bacterial growth (23).

NET function is typically a “double-edged sword”: on one hand, they effectively trap and kill microorganisms, limit their spread, and sequester toxic substances to mitigate tissue damage; on the other hand, excessive or dysregulated NET formation exacerbates tissue injury and inflammatory responses. In IBD, NETs contribute to disease progression through mechanisms such as disrupting the epithelial barrier, activating immune cells, and promoting thrombosis (detailed in sections 3.1 and 3.3) (21, 24, 25). Significantly elevated levels of NET-specific markers (e.g., citrullinated histone H3Cit, MPO-DNA complexes) are detectable in intestinal biopsy tissues and serum from UC mice and patients, and their levels positively correlate with disease activity, providing direct evidence for the pathogenic role of NETs in IBD (26, 27).

### 2.3 Active immune regulators

Recent research continuously updates the understanding of neutrophil functions. Beyond classical antimicrobial roles, neutrophils are important immunoregulatory cells, influencing the functions of other immune cells through direct cell-cell contact and secretion of soluble factors.

In terms of cytokine production, neutrophils exhibit remarkable diversity. They can produce pro-inflammatory cytokines like Tumor Necrosis Factor (TNF)-α and Interferon (IFN)-γ, and under certain conditions, also produce anti-inflammatory cytokines like Interleukin (IL)-10 (28). This functional plasticity allows neutrophils to adjust their immunoregulatory properties based on environmental cues.

**Figure 1:**
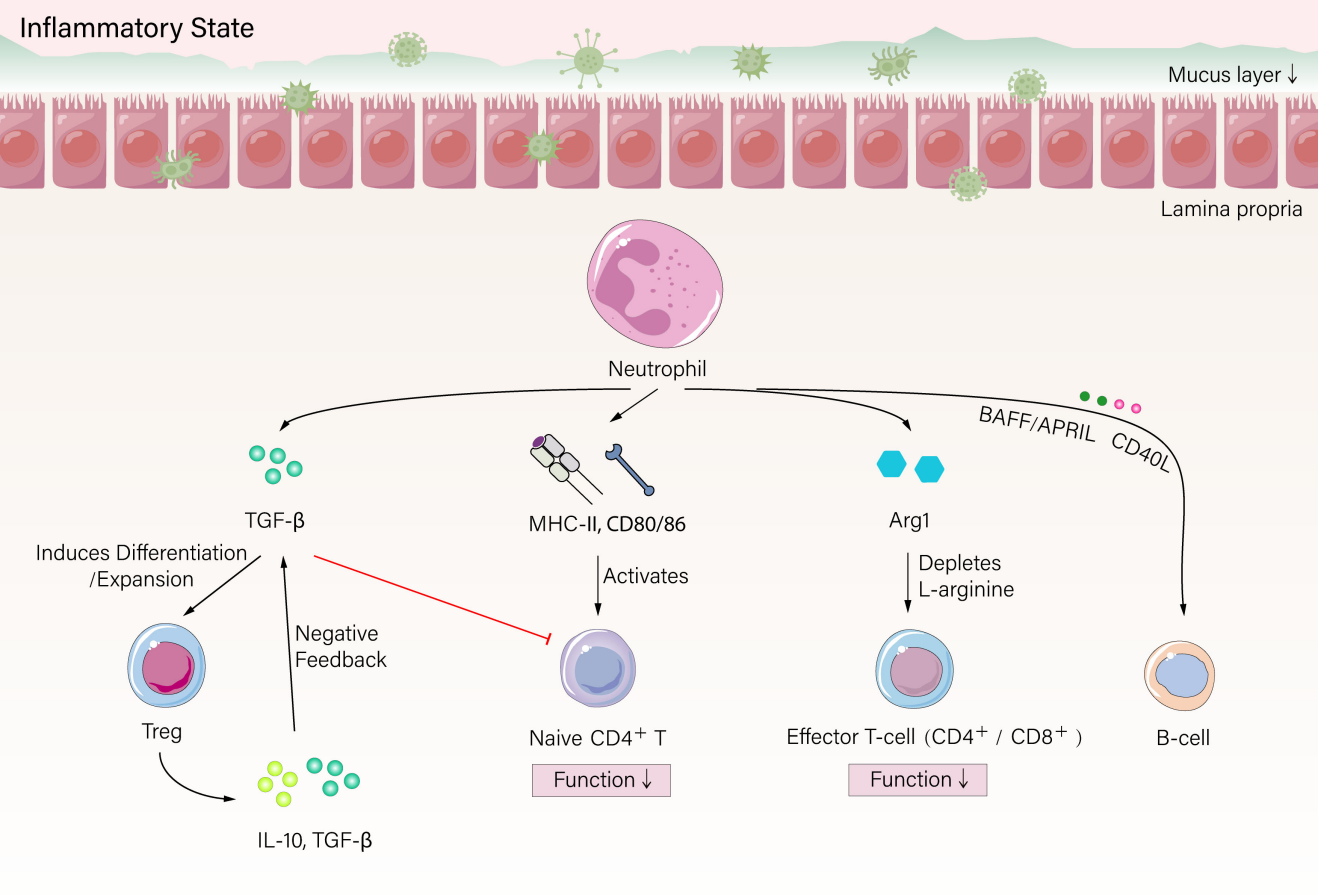
Neutrophils orchestrate a complex immunoregulatory network with T cells, B cells, and T regulatory (Treg) cells in ulcerative colitis. Neutrophils can directly activate naïve CD4^+^ T cells via antigen presentation (MHC-II/CD80/86) while simultaneously suppressing T cell receptor signaling by secreting arginase-1 (Arg1), which depletes local L-arginine. Concurrently, neutrophils provide key survival and class-switching signals to B cells via BAFF, APRIL, and CD40L. A pivotal additional pathway demonstrates that neutrophils also contribute to immune regulation by secreting factors like TGF-β, which promote the differentiation and expansion of Tregs. These induced Tregs subsequently secrete anti-inflammatory cytokines (IL-10, TGF-β), establishing a negative feedback loop that suppresses both neutrophil activation and effector T cell function.

Interactions between neutrophils and adaptive immune cells constitute a crucial pillar bridging innate and adaptive immunity. In regulating T cell responses, neutrophils play a dual role: on one hand, they can directly activate naive CD4^+^ T cells through antigen presentation (expressing MHC class II molecules and costimulatory molecules CD80/86) (29); on the other hand, they can inhibit T cell receptor (TCR) signaling and impede T cell proliferation by secreting arginase 1 (Arg1), which depletes local L-arginine in the microenvironment (30). In interactions with B cells, neutrophils are confirmed as important “helper” cells for B cells, providing critical signals for B cell survival, proliferation, and class-switch recombination by secreting B cell-activating factor (BAFF), a proliferation-inducing ligand (APRIL), and membrane-expressed CD40L (31). In specific locations like the spleen and intestine, neutrophils can even form a “B cell-helper neutrophil” subset, playing important roles in germinal center reactions and efficient antibody production (**Figure 1**).

### 2.4 Context-dependent roles: neutrophil heterogeneity across chronic inflammatory diseases

It is important to emphasize that the roles of neutrophils are not monolithic but are critically shaped by the specific tissue microenvironment. While neutrophils are key players in a spectrum of chronic autoinflammatory diseases, their functional contributions exhibit significant disease-specific characteristics. For instance, in rheumatoid arthritis (RA), synovial fluid neutrophils are pivotal in driving cartilage and bone destruction primarily through the release of proteases like MMP-8 and MMP-9, and via the formation of inflammatory NETs that citrullinate autoantigens, fueling autoimmune responses (32). In psoriasis, skin-infiltrating neutrophils form Munro’s microabscesses and contribute to epidermal hyperplasia through the release of IL-17 and other mediators, within a cytokine milieu dominated by IL-23/Th17 signaling (33). In contrast, within the unique landscape of IBD, neutrophil functions are imprinted by the gut-specific context: an exceptionally high microbial burden, a single-layer epithelial barrier under constant renewal, and a distinct set of tissue-derived signals (e.g., epithelial-derived chemokines like IL-8 and ENA-78 as noted in section 2.2). This culminates in disease-defining pathologies such as crypt abscesses and a direct, continuous interplay with the commensal microbiota—a feature less prominent in sterile or extra-intestinal inflammatory sites. Therefore, the subsequent discussion on neutrophil recruitment, pathogenic mechanisms, and reparative functions in this review will focus on these IBD-specific alterations and their consequences, acknowledging that therapeutic strategies targeting neutrophils may need to be tailored to the organ and disease context.

## 3 Chemotaxis and infiltration: the path of neutrophil accumulation in the IBD mucosa

During IBD development, the recruitment of neutrophils to the intestinal mucosa is a multifactorial, complex process. It begins with local tissue-derived chemotactic signals, followed by neutrophils executing a series of precise molecular events to migrate from the vascular lumen to the tissue interstitium.

### 3.1 Complex chemotactic signaling network

The chemokine network in the intestinal mucosa plays a central role in IBD pathogenesis. Epithelial cells, immune cells, and stromal cells produce various chemokines under inflammatory stimulation, forming chemical gradients that guide neutrophil migration (11). The expression of these chemokines is regulated by key signaling pathways involving transcription factors like NF-κB and STAT3, and microbial products and cytokines can further amplify these signals (34).

Beyond classical chemokines, newly discovered chemotactic mediators have garnered recent attention. For example, formyl peptide receptors (FPRs) and their ligands play important roles in intestinal inflammation (35). These receptors can recognize bacterial-derived formyl peptides and also respond to formyl peptides released by host mitochondria, potentially representing another important mechanism for neutrophil recruitment during tissue damage (36).

Lipid mediators are also crucial in the chemotactic process, constituting a rapid response system for neutrophil recruitment. Leukotriene B4 (LTB4) is one of the most potent neutrophil chemoattractants, produced by activated macrophages, neutrophils themselves, and epithelial cells via the 5-lipoxygenase (5-LOX) pathway (37). By binding to its high-affinity receptor BLT1, LTB4 not only induces strong chemotactic responses and neutrophil aggregation but also enhances integrin affinity, promoting firm adhesion (38). LTB4 production requires 5-lipoxygenase-activating protein (FLAP) to recruit 5-LOX to the nuclear membrane, providing a potential drug target for intervening in LTB4 signaling (39). FLAP inhibitors have shown anti-inflammatory effects in preclinical models (40). Besides LTB4, other lipid mediators like platelet-activating factor (PAF) and the chemerin LXA4 also finely regulate neutrophil migration and retention through their respective receptors (41).

### 3.2 Multifaceted regulation by tissue-derived factors: beyond chemotaxis

It is crucial to recognize that tissue-derived signals in the IBD mucosa exert pleiotropic effects on neutrophils, extending far beyond chemotactic recruitment. Key epithelial- and stromal-derived factors, such as the chemokines IL-8 (CXCL8) and ENA-78 (CXCL5) (42), are abundantly produced during active inflammation. While they establish crucial chemotactic gradients, their role is not limited to guiding neutrophil migration. Upon neutrophil arrival, the sustained high local concentrations of these mediators engage their cognate receptors (e.g., CXCR1/2), triggering intracellular signaling cascades that profoundly influence neutrophil effector functions and lifespan. For instance, signaling through these receptors can activate pro-survival pathways like PI3K/Akt, delaying apoptosis and thereby prolonging the neutrophil presence and activity within the inflamed tissue (43). Similarly, lipid mediators like LTB4 not only are potent chemoattractants but also enhance neutrophil activation, adhesion, and the release of granular enzymes and ROS (44). This multidimensional regulation means that the same tissue-derived factors that recruit neutrophils concurrently “prime” them for enhanced pro-inflammatory activity and extend their functional window. Consequently, therapeutic strategies aimed at modulating the expression or activity of these upstream mediators (e.g., via inhibitors of key synthesis enzymes like 5-LOX/FLAP for LTB4, or receptor antagonists for CXCR1/2) hold significant promise for normalizing neutrophil recruitment, activation, and persistence, offering a strategic point of intervention in IBD.

### 3.3 “Accelerators” and “Brakes” in migration

The neutrophil migration process is coordinately regulated by precise networks of positive and negative signals to balance effective host defense and excessive tissue damage. On the “accelerator” side, besides classical chemokines, certain proteases significantly promote neutrophil recruitment through non-chemotactic mechanisms. For instance, matrix metalloproteinases (MMPs, particularly MMP-9) not only degrade the basement membrane and extracellular matrix, creating physical pathways for cell migration (45), but also proteolytically cleave and activate pre-stored pro-chemokines present in the matrix, such as IL-8/CXCL8, thereby amplifying chemotactic signals locally and forming a positive feedback loop (46).

Regarding the “brake” systems, the body has evolved multiple intrinsic negative feedback mechanisms to prevent uncontrolled inflammation. Among these, Monocyte Chemoattractant Protein-Induced Protein (MCPIP) 1 is a key negative regulator discovered recently. As a ribonuclease, it directly degrades a range of inflammation-related mRNAs (e.g., IL-6, IL-1), limiting the intensity and duration of the inflammatory response at the post-transcriptional level (47). Anti-inflammatory cytokines like IL-10 broadly suppress the gene expression of many pro-inflammatory cytokines and chemokines by activating the STAT3 signaling pathway (48). During the later stages of inflammation, specialized pro-resolving mediators (SPMs) such as lipoxin A4 (LXA4) and resolvin D1 (RvD1) are synthesized. They signal “stop” through their specific G-protein coupled receptors, actively inhibiting further neutrophil infiltration and activation, promoting their apoptosis and clearance, thereby initiating the inflammation resolution program (detailed in section 4.3) (49).

### 3.4 Crossing barriers: transendothelial and transepithelial migration

Neutrophil transmigration across the vascular endothelium is a multi-step, precise process. First, selectin-mediated rolling slows down neutrophils, allowing transient contact with endothelial cells (50). Subsequently, chemokines activate integrins, mediating firm adhesion (51). Finally, neutrophils complete extravasation by finding cell junctions or directly traversing through endothelial cell bodies (52).

Transepithelial migration is more challenging, requiring neutrophils to cross without severely compromising the epithelial barrier function. This process involves dynamic reorganization of epithelial cell junctions and the coordinated action of various adhesion molecules (e.g., ICAM-1, CD47) (53). Recent studies have found that epithelial cells play an active guiding role in this process, rather than being a passive barrier. Upon receiving inflammatory signals from the stromal side, epithelial cells polarize and extend ICAM-1-rich membrane projections, forming “migration pockets” that provide directional guidance and temporary shelter for neutrophils (54). Simultaneously, tight junctions and adherens junctions between epithelial cells undergo reversible rearrangement, creating temporary channels for neutrophil passage (55). This cell-led active migration mode minimizes the increase in epithelial barrier permeability, representing a delicate balance achieved by the host between clearing luminal pathogens and maintaining barrier integrity.

### 3.5 Fate shift in the inflammatory environment: delayed apoptosis

In the inflammatory environment, neutrophil lifespan is significantly extended, which is crucial for the maintenance of chronic inflammation (56). Various inflammatory mediators inhibit neutrophil apoptosis through different signaling pathways. For example, G-CSF promotes the expression of the survival protein Mcl-1 via the JAK2-STAT3 pathway (57); GM-CSF inhibits the activation of the pro-apoptotic protein Bax through the PI3K-Akt pathway (5).

Besides classical cytokines, certain microbial products can also influence neutrophil lifespan. Lipopolysaccharide (LPS) delays apoptosis via Toll-like receptor (TLR) 4 signaling (23), while some metabolites like succinate exert similar effects by activating the GPR91 receptor (58). These findings suggest that the microbiota may influence intestinal inflammation by modulating neutrophil lifespan.

## 4 The dark side: pathogenic mechanisms of neutrophils in IBD

**Figure 2:**
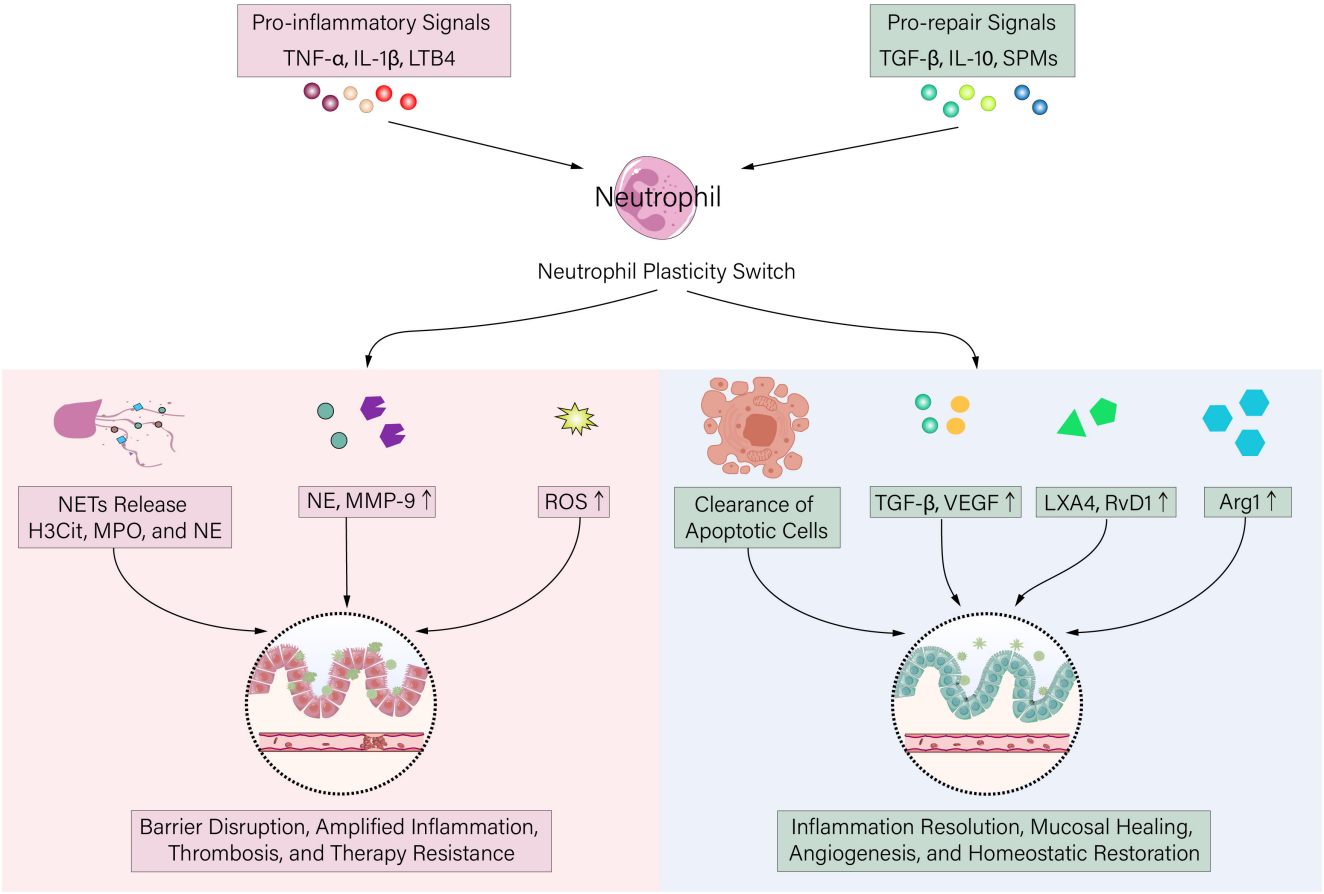
The dual roles and functional balance of neutrophils in inflammatory bowel disease (IBD). This schematic illustrates the dual functions of neutrophils in IBD, whose role is dynamically regulated by signals from the inflammatory microenvironment. Core Mechanism (Center): The balance between pro-inflammatory signals and pro-repair/pro-resolution signals determines the direction of the neutrophil’s “functional plasticity switch”. The Dark Side (Left): When pro-inflammatory signals dominate, neutrophils act as destroyers by releasing NETs, proteases, and ROS, leading to barrier damage, amplified inflammation, and thrombosis. The Light Side (Right): When pro-repair signals dominate, neutrophils act as guardians by clearing debris, secreting repair factors, and pro-resolving mediators, thereby driving inflammation resolution, mucosal healing, and homeostasis restoration. Arrows indicate the dynamic transition paths of neutrophil functional states in response to changes in the microenvironment.

Under IBD pathological conditions, neutrophil function becomes dysregulated, transforming from protective immune cells into major effector cells, causing tissue destruction. This functional shift involves multi-layered mechanisms that collectively drive disease progression (**Figure 2**).

### 4.1 The barrier disruptor

Disruption of intestinal epithelial barrier function is a core pathological feature of IBD, and neutrophils play a key destructive role through both physical compression and biochemical pathways. Firstly, the massive aggregation of neutrophils in the lamina propria and crypts forms crypt abscesses; this physical mass effect directly disrupts the structural integrity of the epithelium (59). Biochemically, various proteases released by neutrophils are the direct “executors” causing barrier damage (60). Among them, NE and matrix metalloproteinase-9 (MMP-9) are particularly important. NE can directly cleave and destroy key epithelial junction proteins, namely Occludin, ZO-1 (tight junctions), and E-cadherin (adherens junctions), thereby increasing epithelial permeability (61–63). MMP-9 degrades type IV collagen, a major component of the basement membrane, destroying the anchoring and support structure for epithelial cells (4).

Beyond directly damaging cellular junctions, neutrophils can also induce epithelial cell death. Recent studies found that myeloperoxidase (MPO) derived from neutrophils can, under specific conditions, catalyze the production of excessive oxidants, inducing mitochondrial dysfunction and initiating programmed necrosis (necroptosis) in epithelial cells (64). This form of cell death leads to the leakage of cellular contents, strongly activating the immune system and further amplifying inflammation. Oxidative stress is another core mechanism. Activated neutrophils produce large amounts of ROS; these highly reactive molecules can not only directly oxidatively damage lipids, proteins, and DNA in epithelial cells (65) but also act as important signaling molecules, oxidatively modifying and inactivating key signaling proteins like protein tyrosine phosphatases (PTPs) (66). Inactivation of PTPs leads to persistent activation of growth factor receptor signaling pathways (e.g., epidermal growth factor receptor (EGFR)), disrupting the normal balance of epithelial cell proliferation, differentiation, and apoptosis, and hindering effective repair processes (67).

### 4.2 Amplifier of the inflammatory storm and driver of chronicity

Neutrophils amplify and sustain the inflammatory response through multiple mechanisms. Firstly, they produce abundant pro-inflammatory cytokines like TNF-α and IL-6, which not only exert direct pro-inflammatory effects but also induce other cells to produce more inflammatory mediators (68). Secondly, neutrophils establish chemotactic gradients by producing chemokines, continuously recruiting more immune cells to the inflammatory site (11).

Recent research has revealed that neutrophils can establish complex positive feedback loops, self-sustaining and amplifying the inflammatory response. For instance, neutrophil-derived IL-1β potently induces epithelial and stromal cells to produce more G-CSF and Granulocyte-Macrophage (GM)-CSF (69). These CSFs not only feed back to the bone marrow, accelerating neutrophil production and release, but also, locally at the inflammatory site, strongly inhibit neutrophil apoptosis via the JAK-STAT and PI3K-Akt pathways, prolonging their lifespan and enhancing their pro-inflammatory functions (70). Neutrophil-derived proteases (e.g., NE) can proteolytically activate pro-IL-1β in macrophages, further amplifying IL-1 signaling (60). Such self-driven inflammatory circuits prevent the spontaneous resolution of initial acute inflammation and represent an important mechanism contributing to the chronicity and relapse of IBD.

### 4.3 NETs: the bridge connecting inflammation and thrombosis

The role of NETs in IBD pathogenesis is increasingly recognized. NETs act as a link tightly connecting local mucosal inflammation with systemic complications like thrombosis. Regarding the promotion of chronic inflammation, the role of NETs is multifaceted (71). The histone components (especially citrullinated histone H3) within the NET scaffold are inherently cytotoxic, directly damaging epithelial and endothelial cell membranes and inducing cell death (11). More importantly, NETs, by virtue of their components HMGB1 and DNA, are typical damage-associated molecular patterns (DAMPs) that can be recognized by pattern recognition receptors (e.g., TLR4, TLR9) (72), persistently activating immune cells like macrophages and dendritic cells to produce pro-inflammatory cytokines such as IL-1β and TNF-α, thereby forming self-reinforcing inflammatory loops that hinder normal inflammation resolution (73).

Regarding thrombosis, NETs provide a highly efficient pro-coagulant scaffold. Their negatively charged DNA backbone efficiently binds and activates coagulation factor XII, the initiator of the intrinsic coagulation pathway, converting it to XIIa and thereby initiating the coagulation cascade (74). Simultaneously, histones in NETs can directly activate platelets, promoting their aggregation, and can damage endothelial cells, converting them from an anticoagulant to a procoagulant phenotype. Crucially, histones can also directly inhibit the activity of activated protein C (APC), which has potent anticoagulant functions (75, 76). Importantly, therapeutic attempts to degrade NETs, such as with DNase, may have paradoxical effects. While intended to dismantle the pro-thrombotic scaffold, DNase treatment can liberate embedded cytotoxic proteins (e.g., histones, NE) from NETs, potentially amplifying microvascular injury in the inflamed intestine, highlighting the complex dual nature of NETs as both a target and a source of pathology (77). These mechanisms act synergistically, making inflammation-rich sites (e.g., mesenteric vessels in IBD patients) prone to microthrombus formation. This not only exacerbates local tissue ischemia and injury but also explains the significantly increased risk of venous thromboembolism in IBD patients.

Moreover, the systemic impact of intestinal NETs extends beyond the vasculature. Recent evidence indicates that neutrophils activated in the inflamed gut can migrate to distant organs, such as the lungs, where their subsequent release of NETs exacerbates tissue damage, illustrating a direct mechanistic link between intestinal inflammation and extra-intestinal complications like pneumonia (78).

### 4.4 The “Mastermind” behind treatment resistance

The mechanisms by which neutrophils contribute to treatment resistance are complex and varied. Regarding glucocorticoid (GC) resistance, besides the known upregulation of the transcription factor Twist1, antagonizing glucocorticoid receptor (GR) transcriptional activity (79), new evidence reveals that neutrophil-derived microvesicles (MVs) play a key role in transmitting resistance (80). These MVs, as important intercellular communication vehicles, can deliver their cargo rich in pro-inflammatory molecules to target cells, thereby creating and maintaining a local microenvironmental inflammatory state unfavorable for GC action.

Regarding resistance to biologics, neutrophils may also play a significant role. Evidence suggests that in the high-protease environment of the inflamed gut, neutrophil-derived serine proteases, including NE and cathepsin G, can proteolytically cleave therapeutic monoclonal antibodies, exemplified by anti-TNFα agents, leading to antibody degradation, inactivation, or the formation of anti-drug antibodies (ADA), significantly reducing their bioavailability and efficacy (81).

The mechanistic link is rooted in the well-documented proteolytic landscape of the inflamed IBD mucosa, which is dominated by neutrophil-derived serine proteases such as neutrophil elastase (NE) and cathepsin G. It is a compelling and plausible hypothesis that these abundant and potent enzymes contribute to the degradation or inactivation of protein-based therapeutics, including anti-TNFα monoclonal antibodies (e.g., infliximab, adalimumab), within the intestinal lumen and mucosal tissue. This proteolytic activity could directly reduce drug bioavailability and potentially generate protein fragments that enhance immunogenicity. Critically, this hypothesis aligns with the clinical observation that a high inflammatory burden—a proxy for intense neutrophil infiltration and protease release—is a consistent predictor of poorer primary response to anti-TNF therapy (82). Thus, while direct in-human evidence of specific antibody cleavage remains to be fully elucidated, neutrophil-mediated proteolysis stands as a key, mechanistically grounded axis of biologic treatment resistance that warrants further investigation.

## 5 The bright side: the protective role of neutrophils in intestinal repair

Despite their significant pathogenic role in IBD onset, neutrophils are also indispensable participants in the tissue repair process. Understanding the protective functions of neutrophils during repair is crucial for developing balanced therapeutic strategies.

### 5.1 The indispensable “Scavenger”

The clearance function of neutrophils is the cornerstone of their protective role, targeting not only invading pathogens but, more critically, the timely removal of apoptotic cells and cellular debris. This “scavenger” function is essential for the timely resolution of inflammation. After fulfilling their duties, large numbers of neutrophils undergo apoptosis. Subsequently, infiltrating or tissue-resident neutrophils and macrophages efficiently phagocytose these apoptotic cells (83). This process is not merely “garbage disposal” but an active anti-inflammatory signal switching event. Upon phagocytosing apoptotic cells, phagocytes receive “find-me” signals (e.g., CX3CL1) and “eat-me” signals (e.g., phosphatidylserine) exposed on the apoptotic cell membrane (84), leading to upregulated secretion of anti-inflammatory factors IL-10 and transforming growth factor (TGF)-β, and downregulated production of pro-inflammatory factors like TNF-α and IL-12, thereby promoting the transition of the inflammatory microenvironment towards the repair phase (85). Neutrophil phagocytic function is highly selective; by recognizing phosphatidylserine (PS) specifically exposed on the surface of apoptotic cells, they avoid mistakenly damaging healthy cells with normal surface charge, ensuring precision and safety in the clearance process (86).

### 5.2 The “Engineer” of tissue repair

During the tissue repair phase, neutrophils transform into active “engineers,” guiding the reconstruction process by secreting a series of growth factors and cytokines. Vascular endothelial growth factor (VEGF) is one of the most important factors. Neutrophil-derived VEGF not only strongly promotes angiogenesis by binding to its receptor VEGFR2, bringing essential oxygen and nutrients to the repairing tissue, but also acts directly on intestinal epithelial cells in a paracrine manner, stimulating their proliferation and migration, making it a key driver of epithelial healing (87). TGF-β produced by neutrophils plays multiple roles: it can suppress excessive inflammation, potently stimulate fibroblast differentiation into myofibroblasts, and promote the synthesis and deposition of extracellular matrix (ECM) components like collagen and fibronectin, providing the structural framework for tissue repair (88).

### 5.3 The “Transmitter” of pro-repair signals

A key function of neutrophils in tissue repair is acting as important mediators of intercellular communication (89). The lipid mediators they secrete are central to actively initiating the inflammation resolution program. Particularly noteworthy are the specialized pro-resolving mediators (SPMs), including resolvins, protectins, and lipoxins (90). These lipid mediators are not mere immunosuppressants but “active regulators” of resolution. For example, lipoxin A4 (LXA4) signals “stop” through its receptor ALX/FPR2, inhibiting neutrophil infiltration; RvD1, via the GPR32 receptor, enhances β-catenin stability in epithelial cells, activating the Wnt/β-catenin pathway and promoting mucosal healing (91, 92). Exogenous administration of RvD1 significantly accelerates mucosal healing in intestinal inflammation models (93). Besides lipid mediators, hepatocyte growth factor (HGF) secreted by neutrophils also plays an important role in promoting cell regeneration and angiogenesis by activating its receptor c-Met and downstream MAPK and PI3K-Akt signaling pathways (94).

### 5.4 The “Coordinator” of cellular collaboration

Another important function of neutrophils in tissue repair is acting as “coordinators,” constructing and regulating a cell microenvironment conducive to repair. Direct interaction with epithelial cells is central to their coordinating role. Research has found that at the edges of mucosal injury, neutrophils, through surface-expressed β2 integrins (e.g., Mac-1), bind to epithelial ICAM-1, not only delay their own apoptosis but also trigger pro-survival and proliferative signals (e.g., Akt and β-catenin pathways) within epithelial cells (95). This contact-dependent signaling directly accelerates wound closure. More strikingly, this interaction can induce epithelial cells to produce and release amphiregulin, a high-affinity ligand for the EGFR, which is crucial for epithelial repair and barrier function restoration (96).

Collaboration with macrophages exemplifies the “baton pass” between immune cells. Neutrophils promote macrophage polarization towards the repair-associated M2 phenotype by secreting factors like lactoferrin and annexin A1 (Annexin A1) (97). These “educated” M2 macrophages subsequently produce large amounts of IL-10, TGF-β, and VEGF, further suppressing residual inflammation and promoting tissue remodeling (98). This functional relay from neutrophils (early cleanup and signal initiation) to macrophages (sustained anti-inflammation and fine-tuned repair) ensures a smooth and efficient transition from the inflammatory phase to the repair phase.

### 5.5 The “Engineer” of metabolic regulation

Recent research reveals the role of neutrophils in reshaping the local metabolic microenvironment during tissue repair, acting as metabolic “engineers.” During the repair phase, neutrophils can alter local energy and substance metabolism by secreting specific metabolic enzymes, creating favorable conditions for tissue repair. A typical example is Arg1. Neutrophil-derived Arg1 catalyzes the hydrolysis of L-arginine, producing ornithine and urea (99). This reaction has dual significance: on one hand, it consumes local L-arginine, thereby limiting substrate availability for inducible nitric oxide synthase (iNOS) and reducing the production of cytotoxic NO (100); on the other hand, the generated ornithine is a precursor for polyamine synthesis (e.g., putrescine, spermidine), which are crucial for cell cycle progression, proliferation, and differentiation (101).

### 5.6 The “Guardian” of microbial homeostasis

In the unique environment of the gut, neutrophils also indirectly promote tissue repair by maintaining microbial homeostasis. Beyond directly clearing pathogenic bacteria, neutrophils shape the composition of the gut microbiota by secreting antimicrobial peptides like α-defensins and cathelicidin (LL-37) (102). These antimicrobial peptides possess selective antibacterial activity, inhibiting the growth of pathogens while relatively sparing beneficial bacteria.

Some neutrophil-derived antimicrobial peptides also have direct immunomodulatory functions. For instance, LL-37 not only has antimicrobial activity but also promotes epithelial cell migration and angiogenesis (5) and influences adaptive immune responses by modulating dendritic cell and T cell functions (103, 104). This pleiotropic functionality allows neutrophils to directly promote tissue repair processes while clearing pathogens.

In summary, neutrophils play multi-dimensional, multi-layered protective roles in the intestinal repair process. From initial inflammation cleanup to subsequent promotion of cell proliferation, neutrophils employ various mechanisms to ensure the restoration of tissue homeostasis. The proper execution of these functions requires precise spatiotemporal regulation; dysregulation at any step may lead to impaired repair or pathological outcomes like fibrosis. Therefore, when formulating IBD treatment strategies, carefully considering how to balance the pathogenic and protective functions of neutrophils is paramount. Future research should focus on deciphering the molecular switches controlling these functional transitions, aiming to develop precision therapies that selectively inhibit pathogenic functions while preserving protective ones.

## 6 Heterogeneity and plasticity: new insights into neutrophil functional diversity

Traditionally, neutrophils were considered a relatively homogeneous cell population. However, with the development of new technologies like single-cell sequencing, researchers have discovered significant heterogeneity and functional plasticity in neutrophils (105). This diversity exists not only between different individuals but also within the same individual across different tissue microenvironments.

### 6.1 Developmental and maturational heterogeneity

Neutrophil heterogeneity is established during their development in the bone marrow. Studies have found a continuous developmental spectrum of neutrophils in the bone marrow, from promyelocytes, myelocytes, metamyelocytes, to band cells and segmented neutrophils. Cells at different stages not only differ morphologically but also exhibit significant differences in surface markers and functional potential (106, 107). For example, less mature CD10^-^ low-density granulocyte (LDG) neutrophil precursors are released in large numbers into the blood during systemic inflammation; these cells have a greater tendency to form NETs and stronger oxidative burst capacity, but their chemotactic migration and phagocytic functions might be weaker than those of fully mature CD10^+^ LDG neutrophils (108). This developmental stage heterogeneity is an important immune reserve strategy, allowing the body to rapidly mobilize neutrophil subsets with different functional emphases depending on the immune challenge (e.g., acute bacterial infection vs. chronic inflammation).

Importantly, this developmental heterogeneity is not merely a feature of mouse models but is directly observable in human IBD. Peripheral blood from patients with active UC and CD shows an increased frequency of immature neutrophil subsets, such as CD10^-^ low-density granulocytes (LDGs) or CD16dim/CD62Lbright populations, which correlate with elevated systemic inflammatory markers (e.g., CRP) and endoscopic disease activity (109). Single-cell RNA sequencing (scRNA-seq) of intestinal lamina propria cells from IBD patients has further confirmed the presence of a neutrophil developmental continuum within the inflamed mucosa, identifying precursor-like subsets expressing high levels of HCAR3 and CMTM2 that are enriched in areas of severe inflammation (110). These findings solidify the concept that emergency granulopoiesis and the recruitment of immature neutrophils are hallmarks of human IBD pathophysiology.

Under inflammatory conditions, the bone marrow accelerates the release of incompletely mature neutrophils, which possess unique surface marker expression profiles and functional characteristics (111). As an example, the CD10^-^ neutrophil subset appears in large numbers during early inflammation, exhibiting stronger migratory capacity and pro-inflammatory properties (112). This developmental heterogeneity provides a new perspective for understanding the dynamic functional changes of neutrophils in inflammatory states.

### 6.2 Tissue-specific neutrophil subsets

When neutrophils enter different tissues from the circulation, their phenotype and function are further “sculpted” by the local microenvironment, exhibiting tissue specificity. Based on the resident organ, functional emphasis, ranging from bactericidal activity to immunoregulatory properties, and surface receptor expression profiles, researchers have identified functionally specialized neutrophil subsets in locations like the spleen, lung, and tumors (52, 113, 114). In the intestinal mucosa, besides classical pro-inflammatory subsets, there also exist subsets with immunoregulatory functions. To illustrate, subsets expressing CD177 and CD16 can limit excessive inflammation by inhibiting NET formation (115), while subsets expressing CD64 possess potent bactericidal functions, specifically responsible for clearing invading pathogens (116). This tissue-level heterogeneity is the cellular biological basis for neutrophils to adapt to local environments and perform specific tasks.

Evidence for functional subset specialization in humans, though not yet fully mapped at the single-cell level within intestinal tissue, is emerging from sophisticated ex vivo models and spatial analyses. Co-culture studies using intestinal organoids derived from IBD patients with autologous or donor neutrophils have demonstrated that neutrophils can adopt distinct functional phenotypes (e.g., pro-inflammatory vs. tissue-remodeling) in response to epithelial-derived signals (117). Immunohistochemical and spatial transcriptomic analyses of human IBD resection specimens have identified geographical niches where neutrophils expressing high levels of GBP1and FCGR1A colocalize with either damaged crypts or healing edges, implying context-dependent functional roles (110).

### 6.3 Dynamic switching of functional states

Neutrophil functional states are not fixed but exhibit high plasticity, allowing dynamic switching between different stages of inflammation based on environmental signals (118). In early inflammation, infiltrating neutrophils primarily exhibit a strongly pro-inflammatory and bactericidal phenotype (often termed “N1” phenotype), expressing high levels of pro-inflammatory cytokines and chemokines, and possessing strong phagocytic and ROS-producing capabilities. As inflammation resolves and the repair phase begins, the same population or newly recruited cells, driven by local signals (e.g., high levels of TGF-β, IL-10, SPMs, and hypoxic conditions), transition towards an anti-inflammatory and tissue-reparative phenotype (akin to “N2” phenotype) (119). This phenotypic switching involves profound transcriptomic and epigenetic reprogramming. For instance, TGF-β signaling promotes the expression of repair-related genes via the Smad pathway (88); hypoxia-inducible factor HIF-1α, induced by tissue hypoxia, not only enhances cellular glycolysis to adapt to the low-oxygen environment but also regulates a series of genes related to angiogenesis and tissue repair (120). The concept of functional plasticity is strongly supported by observations in human IBD. Transcriptomic and proteomic analyses of neutrophils isolated from the blood and intestinal lavage fluid of patients reveal differential activation states depending on disease phase. During active flare, neutrophils exhibit a hyper-reactive profile with enhanced glycolytic metabolism and upregulation of NETosis-related genes. In contrast, following successful therapy induction of remission, neutrophils show increased expression of genes involved in phagocytosis, lipid mediator synthesis (e.g., for resolvins), and responses to anti-inflammatory cytokines like IL-10 (6).

### 6.4 Methodological considerations and complementary approaches for studying neutrophil heterogeneity

While scRNA-seq has revolutionized our understanding of neutrophil heterogeneity by enabling the unbiased classification of transcriptomic states, it is imperative to acknowledge the intrinsic technical and interpretative challenges of this approach. A primary concern is that the procedures required to isolate neutrophils from tissues (e.g., enzymatic digestion, mechanical dissociation) can significantly alter their transcriptional profile and viability, potentially leading to the selective loss of fragile or adherent subsets (121). Consequently, the analyzed cell population may not fully recapitulate the composition and functional spectrum of neutrophils *in situ* within the complex inflammatory milieu of the IBD mucosa. Furthermore, scRNA-seq provides a high-resolution snapshot of gene expression but often lacks crucial spatial context and information on protein activity and post-translational modifications (122).

Therefore, a critical and integrative approach utilizing complementary technologies is essential to overcome these limitations and obtain a holistic view. Spatiotemporal analysis techniques, such as multiplex imaging (e.g., using Hyperion^®^/Imaging Mass Cytometry, Akoya^®^ PhenoCycler, or MACSima^®^), are powerful tools that bridge this gap (123). These methods allow for the simultaneous detection of numerous proteins within the intact tissue architecture, preserving the spatial relationships between neutrophils, other immune cells, epithelial structures, and microbiota. This enables the direct correlation of neutrophil subsets (defined by protein markers) with specific pathological features (e.g., crypt abscesses, ulcer edges) and the quantification of their functional states *in situ*. By integrating data from scRNA-seq (for deep molecular classification) with multiplex imaging (for spatial validation and functional context) and other modalities like flow cytometry and functional assays, future research can more accurately define the roles of specific neutrophil subsets in IBD pathogenesis and resolution, guiding the development of precise therapeutic strategies.

## 7 Microbiota-neutrophil interactions: a key regulatory axis in gut homeostasis

There exists a close bidirectional interaction between the gut microbiota and neutrophils. This interplay not only influences the maintenance of intestinal immune homeostasis but also plays a significant role in IBD pathogenesis.

### 7.1 Regulation of neutrophil function by the microbiota

The gut microbiota profoundly and finely regulates neutrophil function through its metabolic products and structural components. Short-chain fatty acids (SCFAs), especially butyrate, are important metabolites produced by microbial fermentation of dietary fiber. Butyrate, by inhibiting histone deacetylases (HDACs) within neutrophils, can significantly downregulate the production of various pro-inflammatory cytokines (e.g., TNF-α, IL-6) and chemokines, thereby exerting an anti-inflammatory effect (124). Simultaneously, butyrate can enhance neutrophil phagocytic capacity and MPO-dependent bactericidal function, achieving a “calibrated” effect that suppresses excessive inflammation while preserving host defense capabilities (125). Microbial structural components like lipopolysaccharide (LPS) and flagellin directly activate neutrophils via pattern recognition receptors (e.g., TLRs), regulating their survival, NET formation, and cytokine secretion (126). This continuous, low-level microbial signaling is crucial for maintaining neutrophils in a “primed” state under homeostasis.

### 7.2 Shaping of the microbiota by neutrophils

Neutrophils are not passive recipients of microbial signals; they actively shape the composition and spatial distribution of the gut microbiota through their antimicrobial arsenal. By secreting a diverse array of antimicrobial peptides, from defensins to cathelicidin LL-37, reactive oxygen species, and NETs, neutrophils can selectively inhibit or clear certain pathogens and potential pathobionts, thereby creating ecological space for the colonization and growth of beneficial commensals (127). For example, LL-37 has strong killing activity against many Gram-negative bacteria but lesser effects on some Lactobacillus species (128). However, in the pathological state of IBD, this delicate interaction becomes unbalanced. Specifically, dysregulated neutrophil activity, most notably excessive formation of NETs, becomes a direct driver of microbial dysbiosis. While NETs are evolutionarily designed to immobilize and kill pathogens, in the context of chronic intestinal inflammation, their overproduction and impaired clearance lead to a non-selective bactericidal effect. The extruded chromatin fibers decorated with cytotoxic proteins (e.g., histones, myeloperoxidase, NE) create a micro-environment that is hostile not only to invasive pathogens but also to resident commensal bacteria, thereby reducing overall microbial diversity and disrupting the ecological balance essential for intestinal homeostasis (65, 129). Conversely, neutrophil functional defects (as in patients with chronic granulomatous disease, CGD) fail to effectively control the overgrowth of mucosa-associated pathogens (130, 131). The disrupted microbiota, in turn, releases abnormal stimulatory signals, exacerbating aberrant neutrophil recruitment and activation, forming a vicious cycle that drives disease chronicity (132).

## 8 New therapeutic strategies: prospects for precision medicine targeting neutrophils

Based on the deepened understanding of the complex roles of neutrophils in IBD, researchers are developing a range of targeted therapeutic strategies aimed at precisely modulating harmful neutrophil functions while preserving or enhancing their protective roles.

### 8.1 Specific targeting of pathogenic neutrophil subsets

With the growing recognition of neutrophil heterogeneity, targeting specific pathogenic subsets becomes feasible. By identifying subset-specific surface markers, antibody drugs or cell therapies can be developed to selectively eliminate or inhibit pathogenic neutrophils without affecting other protective neutrophil subsets. For example, specific antibodies targeting pro-inflammatory neutrophil subsets highly expressing CD177 have shown promising therapeutic effects in preclinical studies (6). Such precise targeting can effectively suppress inflammatory responses while minimizing interference with normal immune functions.

### 8.2 Modulating signaling pathways regulating neutrophil function

Targeting specific signaling pathways that regulate neutrophil function is another core strategy for precise intervention. Targeting chemokine receptor pathways is currently one of the most mature strategies. CXCR1 and CXCR2 are key receptors mediating neutrophil recruitment to inflammatory sites like the intestine (133). Several small-molecule antagonists, among them AZD5069 and Navarixin, can reversibly block these receptors, effectively inhibiting excessive neutrophil infiltration. Among them, AZD5069 has been evaluated in clinical trials; although it did not meet all primary endpoints in trials for some inflammatory diseases, it demonstrated inhibitory effects on neutrophil recruitment at the biomarker level, revealing its potential clinical value and challenges (134).

Precisely regulating neutrophil apoptosis and survival signals offers the possibility of “spatiotemporally” controlling the inflammatory response. During the peak of inflammation, promoting the apoptosis of pathogenic neutrophils (especially those with abnormally prolonged lifespan) using small molecule drugs (e.g., CDK inhibitor AT-7519) can accelerate inflammation resolution (135). The widely used clinical biomarker fecal calprotectin is precisely a neutrophil-derived protein, and its levels directly reflect the degree of intestinal neutrophil infiltration, underscoring the dual importance of neutrophils as both therapeutic targets and disease activity monitoring indicators (136).

### 8.3 Microbiome-based intervention strategies

Leveraging the microbiota-neutrophil interaction, developing microbiome-based interventions is another promising direction. Specific probiotic strains, such as certain Lactobacillus and Bifidobacterium strains, have been shown to modulate neutrophil recruitment and activation status (137, 138). The mechanisms may involve interactions between probiotics and intestinal epithelial cells or dendritic cells, thereby regulating the balance of chemokines like CXCL1 and CXCL8, and cytokines such as IL-10 and TGF-β (139). Therapies targeting microbial metabolites may also indirectly influence neutrophil function. For instance, supplementing with prebiotics or probiotics that produce butyrate (e.g., *Faecalibacterium prausnitzii*) can utilize butyrate’s HDAC inhibitory activity to downregulate pro-inflammatory cytokine production in neutrophils while enhancing their phagocytic and ROS-dependent bactericidal functions, achieving a “calibration” rather than suppression of neutrophil function (140). Such microecology-based intervention strategies offer new options for the long-term management of IBD due to their relatively high safety profile.

### 8.4 Applications of cell therapy and tissue engineering

Based on neutrophil heterogeneity and plasticity, researchers are exploring the possibility of “reprogramming” their functions for therapeutic purposes. In the field of cancer immunotherapy, pioneering studies have genetically engineered neutrophils to express chimeric antigen receptors (CAR), enabling them to specifically recognize and kill tumor cells (141). Drawing from this concept, it is theoretically possible to design “engineered neutrophils” that can specifically recognize and clear apoptotic cells or specific pathogens in the IBD mucosa, or “reprogram” them towards a phenotype inclined to secrete pro-repair mediators (e.g., IL-10, TGF-β, RvD1), thereby precisely promoting mucosal healing.

In the field of tissue engineering, neutrophil-derived extracellular vesicles (EVs), particularly exosomes, show great potential as cell-free therapeutics. These nanoscale vesicles are naturally rich in various bioactive molecules from their parent cells, including growth factor mRNAs, microRNAs, metabolic enzymes, and antimicrobial peptides. By isolating and enriching EVs released by neutrophils in a reparative phenotype, one can obtain “smart biomaterials” capable of targeted delivery of pro-repair signals, modulating immune responses, and enhancing epithelial regeneration. These could be used for local enema or targeted delivery to promote mucosal healing and tissue repair while avoiding the potential pro-inflammatory and safety risks associated with live cell therapy (142).

### 8.5 Development of personalized treatment strategies

Considering the functional differences in neutrophils between patients, developing personalized treatment strategies is of great importance. By analyzing the phenotypic and functional characteristics of a patient’s neutrophils, the most suitable treatment plan can be selected for each individual. For example, patients with hyperactivated neutrophils might require stronger immunosuppressive therapy, while those with neutrophil functional deficiencies might need strategies to enhance their immune functions. Such personalized approaches are expected to improve treatment efficacy while reducing side effects.

### 8.6 Clinical translational challenges

While the aforementioned strategies herald a new era of neutrophil-targeted therapy for IBD, their translation into clinical practice faces significant challenges that must be squarely addressed. First, the risk of immunosuppression and infection remains a primary concern. For instance, systemic inhibition of core neutrophil chemotaxis pathways (e.g., via CXCR1/2 antagonists) or pro-survival signals, while effective in reducing inflammation, may compromise the essential host-defense functions of neutrophils, potentially leading to an increased susceptibility to bacterial or fungal infections. This necessitates the development of localized delivery systems or shorter treatment regimens to minimize systemic exposure. Second, the precise identification and targeting of pathogenic subsets *in vivo* is technically and biologically complex. Current definitions of neutrophil subsets often rely on a limited set of surface markers or *in vitro* functional assays, which may not fully capture their dynamic and plastic nature within the human inflammatory milieu. Distinguishing “pathogenic” from “protective” neutrophils in real-time during the course of disease is a formidable task, crucial for avoiding the unintended inhibition of repair-promoting functions. Future work must focus on identifying more robust and stable biomarker panels that define pathogenic subsets across different disease stages and patient populations. Moreover, advancing real-time imaging technologies and patient-derived ex vivo models will be key to validating the functional relevance of these subsets before therapeutic targeting. Overcoming these hurdles will require a concerted effort from basic scientists and clinicians to ensure that the promising paradigm of neutrophil precision medicine can be safely and effectively realized for IBD patients.

## 9 Overall field prospects and fundamental limitations

While Section 7.6 discussed the prominent clinical challenges in translating neutrophil-targeted strategies into practice, this section aims to reflect on the broader landscape of fundamental research limitations and prospects. Moving beyond immediate translational hurdles, we examine the constraints inherent in our current models, technologies, and understanding of neutrophil biology that underpin both mechanistic discovery and therapeutic innovation.

### 9.1 Foundational research bottlenecks

Building upon the translational difficulties outlined earlier, several fundamental research bottlenecks must be overcome to deepen our understanding and enable the next generation of therapies. These include limitations in experimental models, characterization of neutrophil heterogeneity, and methodologies for dynamic analysis. Firstly, limitations in research models are a major challenge. Most current mechanistic studies rely on DSS or TNBS-induced mouse colitis models. While these acute models can simulate some inflammatory features of IBD, they hardly fully recapitulate the complex pathology of human IBD (especially CD), characterized by chronicity, relapse, and transmural inflammation (143). Genetically engineered mouse models provide insights into specific gene functions but often fail to fully mimic the genetic heterogeneity and multifactorial interactions of human disease (144). Therefore, developing animal models that more closely resemble human disease pathology and novel *in vitro* systems like organoid-immune cell co-cultures is key for future research.

Secondly, the understanding of neutrophil heterogeneity is still in its infancy. Although single-cell sequencing technologies have revealed different neutrophil subsets (145), the defining criteria, developmental lineage, functional stability, and specific roles of these subsets in the human body remain unclear (146). For instance, it is not yet clear whether these subsets are pre-determined or represent plastic states “educated” by the local microenvironment. The lack of specific surface markers for distinguishing these subsets limits their precise targeting and functional studies.

Thirdly, there is a lack of dynamic studies. Neutrophil functions are highly spatiotemporally specific, playing different or even opposite roles during the initiation, peak, resolution, and repair phases of inflammation (147). Current research methods are mostly “static” observations, making it difficult to capture their dynamic functional transitions *in vivo*. Developing real-time, *in vivo* imaging techniques and reporter gene mouse models to track neutrophil activity will greatly deepen the understanding of this dynamic process.

Finally, bridging the fundamental translational gap remains a profound challenge. This gap encompasses not only the specific clinical risks and targeting dilemmas discussed in Section 7.6 but also the broader journey of distilling complex biological insights (e.g., neutrophil duality, heterogeneity) into druggable targets, reliable biomarkers, and ultimately, therapies whose safety and efficacy are validated through rigorous clinical trials. This path requires sustained collaboration between basic scientists and clinicians.

### 9.2 Future research directions

Facing these challenges, future research should focus on the following cutting-edge directions:

Technology-Driven Mechanism Elucidation: Utilize multi-omics technologies, including spatial transcriptomics, proteomics, and metabolomics, combined with *in vivo* real-time imaging, to map the interaction networks between neutrophils and epithelial cells, fibroblasts, nerve cells, and the microbiota within the intact tissue microenvironment, thereby systematically revealing the molecular map of their functional regulation.

Focus on Human-Relevant Research: Strengthen in-depth analysis of IBD patient samples, including using single-cell technologies to directly analyze neutrophil subsets in human tissues, establishing patient-derived organoid and immune cell co-culture systems, and conducting targeted clinical cohort studies to validate preclinical findings and discover human-specific pathological mechanisms.

Precision and Intelligence in Targeting Strategies: Future therapeutic strategies should move beyond simple “inhibition” or “activation” towards higher-level, precise modulation. This includes developing smart drug delivery systems that release drugs only upon specific signals at the inflammatory site; exploring bifunctional molecules, such as bispecific antibodies that can both block pathogenic chemokines and deliver pro-repair signals; and utilizing cell therapies to “reprogram” patient neutrophils ex vivo before reinfusion, endowing them with enhanced repair functions rather than destructive capabilities.

Integrating Systems Biology Perspectives: Place neutrophil research within the entire gut immune-neural-microbial-epithelial network for systematic consideration. Understanding how neutrophils respond to and modulate gut nervous system signals and how they interact with microbial metabolites will provide a more comprehensive therapeutic perspective, e.g., developing indirect therapies that modulate neutrophil function by regulating the “gut-brain axis” or “microbiota-gut axis.”

## 10 Summary

In conclusion, neutrophils play a complex and paradoxical central role in IBD, acting as both inflammation “arsonists” and repair “firefighters.” Future research should no longer simplistically view them as a homogeneous population or mere destroyers, but rather as dynamic, heterogeneous, and highly plastic cell populations. By overcoming existing research limitations and embracing new technologies and concepts, we can hope to uncover the “molecular switches” controlling neutrophil functional transitions, ultimately achieving precise guidance of their functions, thereby bringing new, more effective, and safer treatment paradigms to IBD patients. This path of exploration not only concerns IBD treatment but will also deepen our understanding of the immune system’s central role in tissue homeostasis.
